# An *in vivo* invertebrate evaluation system for identifying substances that suppress sucrose-induced postprandial hyperglycemia

**DOI:** 10.1038/srep26354

**Published:** 2016-05-19

**Authors:** Yasuhiko Matsumoto, Masaki Ishii, Kazuhisa Sekimizu

**Affiliations:** 1 Teikyo University Institute of Medical Micology, 359 Otsuka, Hachioji, Tokyo 192-0395, Japan; 2 Laboratory of Microbiology, Graduate School of Pharmaceutical Sciences, The University of Tokyo, 7-3-1 Hongo, Bunkyo-ku, Tokyo 111-0033, Japan

## Abstract

Sucrose is a major sweetener added to various foods and beverages. Excessive intake of sucrose leads to increases in blood glucose levels, which can result in the development and exacerbation of lifestyle-related diseases such as obesity and diabetes. In this study, we established an *in vivo* evaluation system using silkworms to explore substances that suppress the increase in blood glucose levels caused by dietary intake of sucrose. Silkworm hemolymph glucose levels rapidly increased after intake of a sucrose-containing diet. Addition of acarbose or voglibose, α-glycosidase inhibitors clinically used for diabetic patients, suppressed the dietary sucrose-induced increase in the silkworm hemolymph glucose levels. Screening performed using the sucrose-induced postprandial hyperglycemic silkworm model allowed us to identify some lactic acid bacteria that inhibit the increase in silkworm hemolymph glucose levels caused by dietary intake of sucrose. The inhibitory effects of the *Lactococcus lactis* #Ll-1 bacterial strain were significantly greater than those of different strains of lactic acid bacteria. No effect of the *Lactococcus lactis* #Ll-1 strain was observed in silkworms fed a glucose diet. These results suggest that the sucrose diet-induced postprandial hyperglycemic silkworm is a useful model for evaluating chemicals and lactic acid bacteria that suppress increases in blood glucose levels.

Elevated blood glucose levels due to excessive intake of sugar lead to the development and worsening of lifestyle-related diseases such as diabetes^1^,^2^. The increase in the number of type II diabetes patients in the world has become a public health problem^3^. Therefore, suppressing the excessive intake of sugars is important for maintaining healthy lives.

Sucrose is a major sweetener that is added to a variety of foods and beverages^4^,^5^. Excessive intake of sucrose causes postprandial hyperglycemia and the onset of lifestyle-related diseases, such as obesity and diabetes^6^,^7^,^8^. Minimizing dramatic increases in blood glucose levels caused by the intake of excess sucrose can help prevent lifestyle-related diseases. Sucrose decomposes into glucose and fructose by catalytic reaction with α-glycosidase in the intestine and is absorbed through the intestinal membranes, resulting in increased blood glucose levels^9^. Acarbose and voglibose, which are α-glycosidase inhibitors that inhibit the increase in postprandial blood glucose levels, are used as anti-diabetic agents^9^. Therefore, foods containing substances with inhibitory effects against α-glycosidase are expected to suppress the increases in blood glucose levels caused by excessive sucrose ingestion^10^,^11^.

Blood glucose levels after intake of sucrose are regulated by the actions of various organs^10^,^11^. Therefore, evaluation of active substances that inhibit increases in blood glucose levels induced by intake of sucrose requires experiments using whole animals. Conventionally, anti-diabetic compounds are evaluated in mammals, such as mice and rats^12^. The use of a large number of mammals in experiments, however, is costly and associated with ethical issues regarding animal welfare.

We have proposed the silkworm as a useful experimental animal for screening therapeutically effective compounds against infectious diseases and metabolic diseases. A large number of individual silkworms can be reared in a smaller space compared to mammals such as mice^13^. In addition, quantitative injection of drugs into the hemolymph, insect blood, can be performed with a standard 1-ml syringe^14^,^15^. Injection by syringes is difficult in small invertebrates such as nematodes and fruit flies, which are commonly used model animals. We previously reported that silkworms are useful for evaluating the toxicity of candidate drug compounds^16^,^17^,^18^. Moreover, we previously established silkworm-infection models with pathogenic bacteria and fungi pathogenic to humans for evaluation of the therapeutic activities of antibiotics^14^,^19^,^20^,^21^,^22^. Furthermore, silkworms exhibit pharmacokinetic features of various compounds, such as antibiotics and toxic chemicals, similarly to mammals^16^,^19^,^23^. Recently, we have succeeded in identifying a novel antibiotic, Lysocin E, by screening with the silkworm-infection model^22^. Therefore, we have proposed the usefulness of silkworm-infection models for screening therapeutically effective antibiotics^14^,^19^,^21^,^22^. An additional remarkable feature of the silkworm as an experimental animal model is that one can easily collect large amounts of hemolymph for biochemical analyses. Taking advantage of this feature, we established diabetic silkworm models that were useful for evaluating therapeutic activities of agents against both type I and type II diabetes^24^,^25^,^26^. Although we previously reported that glucose levels in the silkworm hemolymph rapidly increase following intake of a glucose-containing diet^24^, we did not examine the effect of excessive sucrose intake.

Acarbose, an α-glycosidase inhibitor, is produced by *Actinoplanes sp.* SE50/110, which is a Gram-positive bacterium^27^. Moreover, increases in the blood glucose levels of mice induced by sucrose ingestion is suppressed by adding *Lactobacillus rhamnosus*, a lactic acid bacterium, to the diet^28^. Furthermore, heat-treated cell fractions of a strain of lactic acid bacterium contain α-glycosidase inhibitory activity^29^. Therefore, we focused on lactic acid bacteria as a candidate organism that inhibits α-glycosidase activity in the intestine.

Here we established a novel silkworm model for evaluating therapeutically effective substances against an increase in blood glucose levels after ingestion of sucrose. Silkworm hemolymph glucose levels rapidly increased after ingesting sucrose. We also demonstrated that the sucrose diet-induced increases in the hemolymph glucose levels were suppressed by adding α- glycosidase inhibitors to the sucrose diet. Furthermore, the increases in the glucose levels were suppressed by adding certain strains of lactic acid bacteria to the sucrose diet. Our findings indicate that the silkworm is a useful experimental animal for investigating the effects of functional lactic acid bacteria to control blood glucose levels after ingesting foods containing sucrose.

## Results

### Intake of sucrose increases glucose levels in the silkworm hemolymph

**Figure 1:**
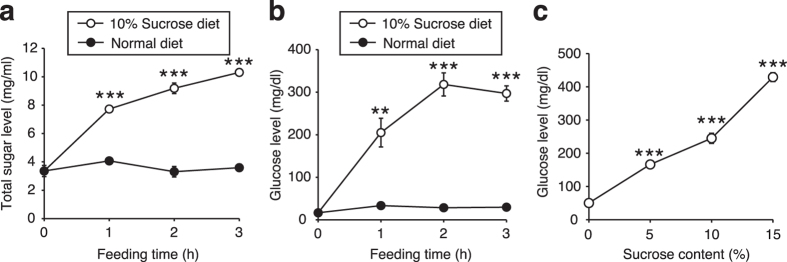
Dietary sucrose-induced increases in total sugar and glucose levels in silkworm hemolymph. (**a**,**b**) Silkworms were fed a normal diet (Normal diet) or a diet containing 10% (w/w) sucrose (10% Sucrose diet). Total sugar and glucose levels in the silkworm hemolymph were measured for 0, 1, 2, and 3 h (n = 4/group). (**c**) Silkworms were fed a normal diet (Normal diet) or a diet containing 5, 10, 15% (w/w) sucrose. Glucose levels in the silkworm hemolymph were measured for 1 h (n = 4/group). Data represent mean ± SEM. Statistically significant differences between groups were evaluated using Student’s *t*-test. **P < 0.01, ***P < 0.001 versus Normal diet.

Sucrose in foods decomposes into glucose and fructose by catalytic reaction with α-glucosidase in the intestine and each sugar is absorbed through the intestinal membranes in mammals^9^. In the present study, we tested whether total sugar and glucose levels in the silkworm hemolymph increase following dietary intake of sucrose. Our findings demonstrated that total sugar and glucose levels in the silkworm hemolymph following ingestion of a 10% sucrose diet immediately increased, whereas no increase was observed in silkworms fed a diet without added sucrose (normal diet; Fig. 1a,b). Moreover, glucose levels in the silkworm hemolymph were increased by addition of sucrose to the diet in a dose-dependent manner (Fig. 1c). Furthermore, thin layer chromatography analysis revealed that sucrose was hardly detectable in the silkworm hemolymph following ingestion of a 10% sucrose diet (Supplementary Fig. 1). These findings suggest that silkworms immediately absorb glucose produced by sucrose upon reaction with α-glucosidase in the intestine.

### Inhibitory effects of acarbose and voglibose against increases in glucose levels in the silkworm hemolymph induced by the intake of sucrose

**Figure 2:**
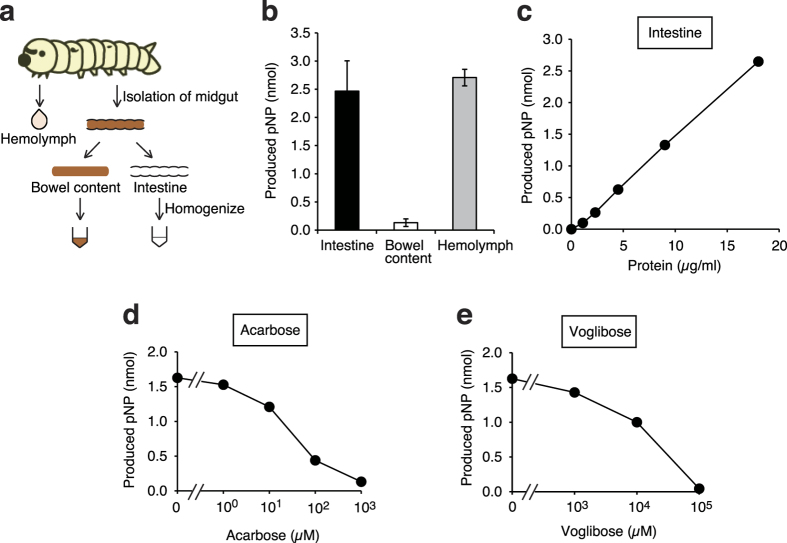
Inhibition of α- glycosidase activity in crude lysate of silkworm intestine by the addition of acarbose or voglibose *in vitro*. (**a**) Scheme for the preparation of samples of silkworm for *in vitro* experiments. Silkworms were fed a normal diet for 1 day. Silkworm hemolymph, bowel content, and intestines were collected. The isolated intestine was disrupted by sonication, and a crude lysate of the intestine was used for analysis. (**b**) The α-glycosidase activity per 1 μg of protein in the silkworm hemolymph, bowel content, and intestines was measured (n = 4–5/group). (**c**) The α-glycosidase activity in the crude lysate with different amounts of silkworm intestine was measured. (**d,e**) The α-glycosidase activity in the crude lysate of silkworm intestine in reaction mixtures with or without acarbose or voglibose was measured. pNP: *p*-nitrophenol.

The silkworm genome contains genes coding homologues of α-glycosidase, which catalyzes the decomposition reaction from sucrose to glucose and fructose^30^. Therefore, we hypothesized that sucrose is degraded by α-glycosidase in the silkworm intestinal tract and is absorbed through the intestinal membranes as in mammals. We found α-glycosidase activity in both the crude lysate of intestine and in the silkworm hemolymph. The specific α-glycosidase activity in crude lysate of the silkworm intestine was much higher than that in the bowel (Fig. 2a–c). Furthermore, the α-glycosidase activity in crude lysate of the silkworm intestine was inhibited by the addition of acarbose or voglibose, which are α-glycosidase inhibitors clinically used for diabetic patients (Fig. 2d,e).

**Figure 3:**
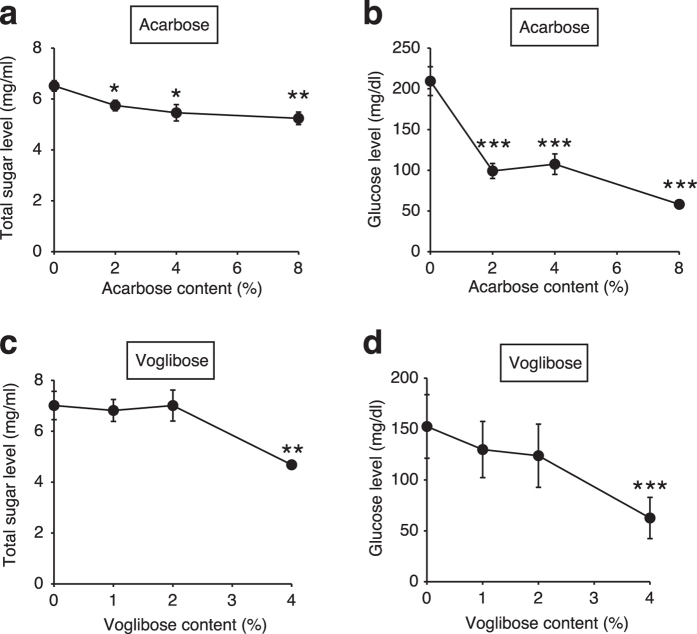
Inhibitory effects of acarbose and voglibose on dietary sucrose-induced increases in glucose levels in silkworm hemolymph. (**a**–**d**) Silkworms were fed a diet containing 10% (w/w) sucrose with or without acarbose or voglibose for 1 h. Total sugar and glucose levels in the silkworm hemolymph were measured (n = 5/group). Data represent mean ± SEM. Statistically significant differences between groups were evaluated using Student’s *t*-test. *P < 0.05, **P < 0.01, ***P < 0.001.

**Figure 4:**
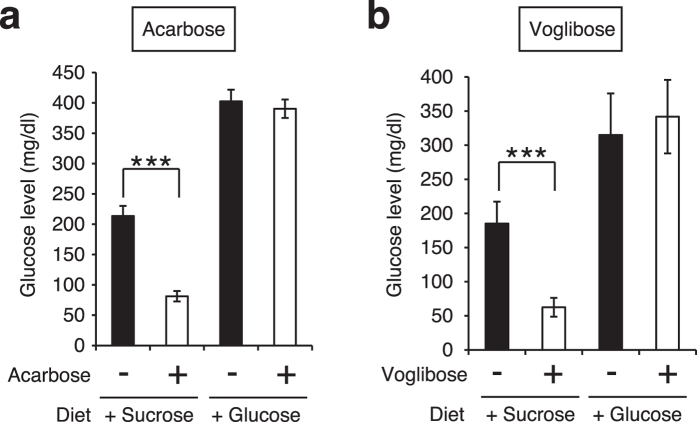
Differences in the inhibitory effects of acarbose and voglibose on dietary sucrose or glucose-induced increases in glucose levels in silkworm hemolymph. (**a,b**) Silkworms were fed a diet containing 10% (w/w) sucrose or glucose with or without acarbose (8% [w/w] in diet) or voglibose (4% [w/w] in diet) for 1 h. Glucose levels in the silkworm hemolymph were measured (n = 5/group). Data represent mean ± SEM. Statistically significant differences between groups were evaluated using Student’s *t*-test. ***P < 0.001.

We next examined whether the increase in silkworm hemolymph glucose levels induced by the intake of sucrose would be suppressed by adding acarbose or voglibose to the sucrose diet. Both total sugar and glucose levels in the silkworm hemolymph following intake of 2%, 4%, or 8% (w/w) acarbose or 4% (w/w) voglibose in addition to a 10% sucrose diet were much lower than those following intake of a 10% sucrose diet alone (Fig. 3). In mammals, acarbose and voglibose inhibit increases in blood glucose levels induced by ingestion of sucrose, but not by ingestion of glucose^31^. We tested whether the effects of these α-glycosidase inhibitors exhibited the same features in silkworms. The glucose levels in the silkworm hemolymph following intake of 8% (w/w) acarbose or 4% (w/w) voglibose in addition to a 10% sucrose diet were lower than those following intake of the sucrose diet, but not intake of a 10% glucose diet (Fig. 4). These findings suggest that acarbose and voglibose have inhibitory effects against increases in glucose levels induced by the ingestion of sucrose by interfering with α-glycosidase activity in silkworms as well as in mammals.

### Effect of lactic acid bacteria against increases in silkworm hemolymph glucose levels induced by intake of sucrose

**Figure 5:**
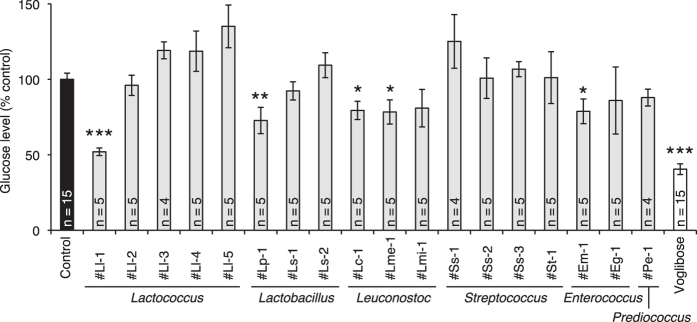
Inhibitory effects of lactic acid bacteria against dietary sucrose-induced increases in silkworm hemolymph glucose levels. Silkworms were fed a diet containing 10% (w/w) sucrose with or without lactic acid bacteria (25% [w/w] in diet) or voglibose (4% [w/w] in diet) for 1 h. Glucose levels in the silkworm hemolymph were measured (n = 4–15/group). Data represent mean ± SEM. Statistically significant differences between groups were evaluated using Student’s *t*-test. *P < 0.05, **P < 0.01. ***P < 0.001.

**Figure 6:**
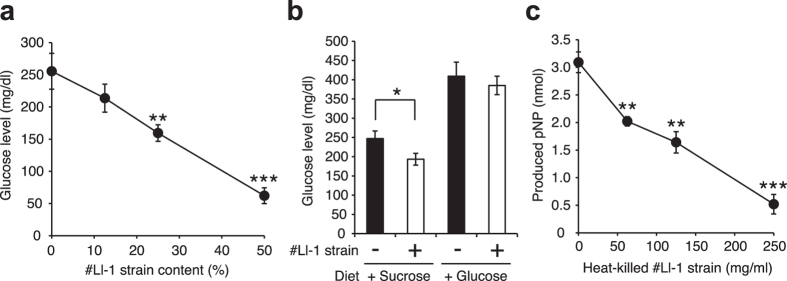
Differences in the inhibitory effects of *Lactococcus lactis* #Ll-1 strain against dietary sucrose or glucose-induced increases in silkworm hemolymph glucose levels. (**a**) Silkworms were fed a diet containing 10% (w/w) sucrose with or without the *Lactococcus lactis* #Ll-1 strain (0–50% [w/w] in diet) for 1 h. Glucose levels in the silkworm hemolymph were measured (n = 5/group). (**b**) Silkworms were fed a diet containing 10% (w/w) sucrose or glucose with or without *Lactococcus lactis* #Ll-1 strain (25% [w/w] in diet) for 1 h. Glucose levels in the silkworm hemolymph were measured (n = 5/group). (**c**) The α-glycosidase activity in the crude lysate of silkworm intestine in reaction mixtures with or without heat-killed *Lactococcus lactis* #Ll-1 strain (0–250 mg/ml) was measured (n = 3/group). pNP: *p*-nitrophenol. Data represent mean ± SEM. Statistically significant differences between groups were evaluated using Student’s *t*-test. *P < 0.05, **P < 0.01. ***P < 0.001.

We next screened lactic acid bacteria from our stock of lactic acid bacteria that inhibit increases in silkworm hemolymph glucose levels induced by the intake of sucrose (Table 1). The addition of some lactic acid bacteria to the sucrose diet inhibited the sucrose-induced increase in glucose levels in the silkworm hemolymph (Fig. 5). The inhibitory effects were dose-dependent in the case of the *Lactococcus lactis* #Ll-1 strain, which had the greatest inhibitory effect (Fig. 6a and Supplementary Fig. 2). On the other hand, the inhibitory effect of the *Lactococcus lactis* #Ll-1 strain against increases in glucose levels in silkworm hemolymph was not observed following intake of a 10% glucose diet (Fig. 6b). In addition, the *in vitro* α-glycosidase activity in crude lysate of the silkworm intestine was inhibited by the addition of heat-killed cells of the *Lactococcus lactis* # Ll-1 strain (Fig. 6c). These findings suggest that effect of the *Lactococcus lactis* #Ll-1 strain to inhibit increases in hemolymph glucose levels was due to suppressed absorption of sugars through the intestinal tract by the inhibition of α-glycosidase in the intestine.

## Discussion

In this study, we established a simple method using silkworms to identify substances suppressing an increase in blood glucose levels caused by ingesting sucrose. In addition, we found that some lactic acid bacteria can inhibit the sucrose-induced increase in hemolymph glucose levels in the silkworm.

Nutrients contained in the diet are absorbed from silkworm intestine to hemolymph and are transferred into the various organs like in mammalian animals. Silkworms have organs such as intestine, fat body, and malpighian tubule, which function for excretion of exogenously administrated chemicals. Moreover, silkworms can maintain glycogen as absorbed carbohydrates in the fat body and the muscle^32^,^33^. Furthermore, insulin-signaling pathway and AMPK pathway in silkworm function to regulate the sugar levels in the hemolymph^24^,^34^. Therefore, the systems for uptake and storage of sugars show common features between silkworms and mammalian animals including humans. On the other hand, the marked difference in silkworms and humans is that the major sugar in hemolymph of silkworms is trehalose, which is composed of two molecules of glucose^33^. Silkworms synthesize trehalose with trehalose synthase expressed in cells of the whole body and the resulting trehalose is released into the hemolymph^35^. We focused on the common features of the absorption mechanism of dietary sugars between silkworm and human.

Sucrose is added as a sweetener to various dishes, sweets, and beverages^4^,^5^. Currently, lifestyle-related diseases such as diabetes, which is due to obesity caused by excessive sucrose intake, are increasing worldwide^6^,^7^,^8^. To prevent high blood glucose levels, diet and exercise therapies seem to be effective. In many cases, however, it is a burden for the patients to continue these therapies^36^. The development of food additives that inhibit the increase in blood glucose levels may be effective for supporting continuous diet therapy. Lactic acid bacteria selected by our evaluation system using silkworms are expected to be effective diet therapy not only for patients with obesity and diabetes, but also for pre-diabetic peoples.

*Lactococcus, Lactobacillus, Leuconostoc*, and *Enterococcus* species have been used in the long history of the production of fermented foods^37^. Among the five strains of *Lactococcus lactis*, only the #Ll-1 strain exhibited effectiveness in the silkworm evaluation system. This finding suggests that there are differences in the α-glycosidase inhibitory effects of lactic acid bacteria strains of the same species. Finding the strains that suppress increases in blood sugar levels requires the use of an evaluation system utilizing an animal model. We propose the use of the silkworm for this purpose. The silkworm system will be highly beneficial for rapid and low-cost selection of useful strains of lactic acid bacteria.

Each isolated strain of *Lactococcus lactis* is known to be different in the ability to utilize arginine, glutamate, and citrate^38^,^39^. The difference of their utilizing abilities causes difference in the amount of metabolite, such as γ-aminobutyric acid (GABA)^40^,^41^. Moreover, the comparative genome analysis among the various strains of *Lactococcus lactis* revealed gene modifications, such as introduction of foreign genes with a plasmid and mutations of endogenous genes^38^. We speculate that the gene modifications in the genome of *Lactococcus lactis* #Ll-1 strain caused alteration in the production of metabolites that have α-glucosidase inhibitory activity.

We found that some lactic acid bacteria strains inhibited an increase in silkworm hemolymph glucose levels induced by the intake of sucrose in a dose-dependent manner (Supplementary Fig. 3). Moreover, we also found that the *Lactobacillus plantarum* #Lp-1 strain, *Leuconostoc carnosum* #Lc-1 strain, and *Enterococcus mundtii* #Em-1 strain inhibited an increase in silkworm hemolymph glucose levels induced by the intake of glucose (Supplementary Fig. 4). We assume that the *Lactobacillus plantarum* #Lp-1 strain, *Leuconostoc carnosum* #Lc-1 strain, and *Enterococcus mundtii* #Em-1 strain have a potential to inhibit the glucose uptake system in silkworm intestine. The inhibitory mechanism by the lactic acid bacteria against glucose uptake remains to be elucidated.

We tested whether α-glucosidase activities of silkworm intestine were decreased by intake of sucrose diet with voglibose or *Lactococcus lactis* #Ll-1 strain. However, we could not detect the inhibition of α-glucosidase activities by the addition of voglibose or *Lactococcus lactis* #Ll-1 strain to the sucrose diet (Supplementary Fig. 5). We speculate that since voglibose or *Lactococcus lactis* #Ll-1 was washed out during the isolation step of silkworm intestine, inhibition of the α-glucosidase activity could not be detected.

To evaluate the therapeutic effectiveness of drugs and foods, mammalian models such as mice and rats, have been used^12^. Screening using a large number of mammals is expensive. Rearing silkworms does not require a large space, and the use of a large number of individuals with low cost is thus possible^13^. Furthermore, from the point of view of animal welfare, experiments with mammals must be carried out in accordance with the 3R concept, i.e. Replacement, Reduction, and Refinement, which is an international principle^42^. Utilizing silkworms as an alternative animal model is consistent with the concept of Replacement. To apply the results obtained by silkworms to humans regarding the therapeutic effects of drugs and functions of foods, further studies with mammals followed by clinical trials with humans are needed. Thus, our findings about the effects of lactic acid bacteria to decrease sugar levels in the silkworm hemolymph should be further examined in mammals and eventually in humans. We consider that the use of silkworms for the screening stage prior to preclinical testing in the development of drugs and foods will reduce the number of mammals used in experiments and decrease costs.

Compounds in chemical libraries are usually provided in small quantity. Therefore, we aimed to establish a screening system that could be performed with small amounts of sample by using minimal number of silkworms per group for evaluation.

Mulberry leaves, a major dietary component for silkworms, includes several types of α-glucosidase inhibitory compounds^43^. Miglitol, a derivative of 1-deoxynojirimycin contained in mulberry leases, is used as an α-glycosidase inhibitor for the treatment of diabetes^44^. We considered that α-glucosidase inhibiting compounds in mulberry leaves might affect the silkworm evaluation system. The artificial diet (Silkmate 2S) used in this study contains as low as 10% mulberry leaf extract in the diet. Therefore, we established an experimental system for evaluating α-glucosidase inhibitory activity using the artificial diet instead of mulberry leaves to reduce the effects of the compounds in mulberry leaves.

We found that the glucose levels in the silkworm hemolymph following intake of 8% (w/w) acarbose or 4% (w/w) voglibose added to a normal diet were lower than those following intake of normal diet (Supplementary Fig. 6). We assume that the degradation of polysaccharide contained in the normal diet is inhibited by acarbose or voglibose.

In conclusion, we established an *in vivo* evaluation system with silkworms that is useful for identifying the components that inhibit increases in blood glucose levels after ingesting sucrose. The lactic acid bacteria identified using this system are expected to be useful for the development of functional foods that help to prevent various lifestyle-related diseases including diabetes.

## Methods

### Silkworm rearing conditions

Silkworms were reared according to the previously reported method^14^,^15^. The eggs of the silkworm (Hu Yo x Tukuba Ne) were purchased from Ehime sericulture incorporated company (Ehime, Japan). Larvae hatched from the eggs were fed an artificial diet (Silkmate 2S, Nihon Nosan Corporation, Tokyo, Japan) and reared to the fifth-instar stage at 25–27 °C. A 10% (w/w)-sucrose or glucose diet was prepared by mixing Silkmate 2S and D-sucrose or D-glucose. A lactic acid bacteria-containing diet was prepared by mixing the 10%-sucrose or glucose diet and pellet (wet weight mg) of lactic acid bacteria harvested by centrifugation from full growth.

### Isolation and culture of lactic acid bacteria

Lactic acid bacteria isolated from natural resources were grown in MRS (de Man, Rogosa, Sharpe) medium containing 0.5% calcium carbonate. Gram-positive bacteria that formed a clear zone around the colonies were identified as lactic acid bacteria. Species of the isolated lactic acid bacteria were determined by sequencing analysis of DNA genes encoding rRNA using previously reported primers^45^. Bacterial species were identified based on ≥99% sequence matching. The lactic acid bacteria were inoculated into MRS medium and cultured at 30 °C or 37 °C for 1–3 days under anaerobic conditions to full growth.

### Chemicals

Acarbose was purchased from LKT Laboratories, Inc. (St. Paul, MN, USA). Voglibose was kindly provided by Takeda Pharmaceutical Company (Osaka, Japan). P-Nitrophenyl-α-D-glucoside (*p*NP-αGlc) was purchased from Sigma (St Louis, MO, USA).

### Determination of sugar in hemolymph

Total sugar levels in the hemolymph were determined by the method described previously^24^. Five microliters of hemolymph was collected from the silkworms through a cut on the first proleg, and immediately mixed with 9 volumes of 0.6 N perchloric acid. The supernatant after centrifugation at 15,000 rpm (20,400 g) for 3 min was appropriately diluted with distilled water for sugar determination. Total sugar in the hemolymph was determined using the phenol-sulfuric acid method^46^. Serially diluted D-glucose solution was used as a standard. Glucose levels in the hemolymph were determined using a glucometer (Accu-Check, Roche).

### Determination of α-glycosidase activity

The α-glycosidase activities in the intestine, bowel content, and hemolymph of the silkworm were determined according to the previously reported method^30^. Silkworms were fed a normal diet for 1 day. The midgut, intestine with bowel content, was excised from the silkworms and the bowel content was isolated from the intestine. The isolated bowel content was mixed with 100 μl phosphate buffered saline (PBS). Intestine was mixed with 250 μl PBS and sonicated using a Sonifier 450A (Branson Ultrasonics Corporation, Danbury, CT, USA). The intestinal crude lysate of intestine was subjected to the enzyme assay as the intestine sample. Twenty microliters of hemolymph was collected from the silkworms, and immediately mixed with an equal volume of PBS. Each sample was added to 100 μl of the reaction mixture, which contained 10 mM *p*NP-αGlc and 100 mM piperazine-1,4-bis(2-ethanesulfonic acid) (pH 6.5), and incubated at 37 °C for 30 min. After incubation, 100 μl of 2 M tris(hydroxymethyl)aminomethane was added to the reaction mixture to terminate the reaction. The absorbance of the product, *p*-nitrophenol, was measured at a wavelength of 405 nm using a Beckman Coulter DU 730 spectrophotometer (Beckman Coulter, Fullerton, CA, USA). The reaction mixture without protein sample was used as a background of the measurement. The amount of *p*-nitrophenol was calculated using the molar extinction coefficient of *p*-nitrophenol (18,200 mol/L/cm).

### Statistical analysis

All experiments were performed at least twice and the data are shown as the mean ± standard error of the mean (SEM). The significance of differences was calculated using a two-tailed Student’s *t*-test at the significance level alpha = 0.05.

## Additional Information

**How to cite this article**: Matsumoto, Y. *et al*. An *in vivo* invertebrate evaluation system for identifying substances that suppress sucrose-induced postprandial hyperglycemia. *Sci. Rep.* **6**, 26354; doi: 10.1038/srep26354 (2016).

## Supplementary Material

Supplementary Information

## Figures and Tables

**Table 1:** List of lactic acid bacteria strains used in this study.

**Strain**	**Species**	**Source**
#Ll-1	*Lactococcus lactis*	Kiwi
#Ll-2	*Lactococcus lactis*	Angleworm
#Ll-3	*Lactococcus lactis*	Slug
#Ll-4	*Lactococcus lactis*	Tree berry
#Ll-5	*Lactococcus lactis*	Tree berry
#Lp-1	*Lactobacillus plantarum*	Bran
#Ls-1	*Lactobacillus sakei*	Kimchi (Korean pickle)
#Ls-2	*Lactobacillus sakei*	Bran
#Lc-1	*Leuconostoc carnosum*	Kimchi (Korean pickle)
#Lme-1	*Leuconostoc mesenteroides subsp. mesenteroides*	Kimchi (Korean pickle)
#Lmi-1	*Leuconostoc miyukkimchii*	Bran
#Ss-1	*Streptococcus salivarius*	Apple
#Ss-2	*Streptococcus salivarius*	Tree leaf
#Ss-3	*Streptococcus salivarius*	Pineapple
#St-1	*Streptococcus thermophilis*	Kiwi
#Em-1	*Enterococcus mundtii*	Soil
#Eg-1	*Enterococcus gallinarum*	Tree leaf
#Pe-1	*Pediococcus ethanolidurans*	Bran

Species of the isolated lactic acid bacteria were determined by sequencing analysis of DNA genes encoding rRNA using previously reported primers^45^. Bacterial species were identified based on ≥99% sequence matching.
