# Supplementary material for: An in vivo invertebrate evaluation system for identifying substances that suppress sucrose-induced postprandial hyperglycemia
Source: Sci Rep. 2016 May 19;6:26354. doi: 10.1038/srep26354 (PMC4872229; doi:10.1038/srep26354)
Supplement: Supplementary Information [file srep26354-s1.pdf]

Supplementary information

An *in vivo* invertebrate evaluation system for identifying substances that suppress sucrose-induced postprandial hyperglycemia

Yasuhiko Matsumoto, Masaki Ishii, and Kazuhisa Sekimizu

[1]*Laboratory of Microbiology, Graduate School of Pharmaceutical Sciences, The University of Tokyo, 7-3-1 Hongo, Bunkyo-ku, Tokyo 111-0033, Japan.*

Correspondence to: Dr. Yasuhiko Matsumoto (ymatsumoto@mol.f.u-tokyo.ac.jp) and Dr. Kazuhisa Sekimizu (sekimizu@mol.f.u-tokyo.ac.jp), Laboratory of Microbiology, Graduate School of Pharmaceutical Sciences, The University of Tokyo, 7-3-1 Hongo, Bunkyo-ku, Tokyo 113-0033, Japan, Tel.: +81-358-414-820, Fax: +81-356-842-973

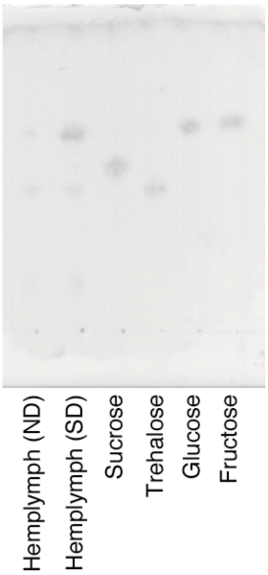

Supplementary figure 1 Thin layer chromatography analysis of sugar content in hemolymph of silkworms made hyperglycemic by intake of a high-sucrose diet for 1 h. Silkworms were fed a normal diet (Normal diet: ND) containing a normal 10% (w/w) sucrose (Sucrose diet: SD) for 1 h. Silkworm hemolymph was collected. The hemolymph (600 μl) was mixed with trichloroacetic acid (TCA) solution (final 5%) and incubated at 4°C for 30 min. The mixture was centrifuged at 15,000 rpm for 3 min and the supernatant was diluted to 4 fold with 5% TCA solution. Sucrose (0.5 mg/ml), trehalose (4 mg/ml), glucose (0.5 mg/ml), and fructose (0.5 mg/ml) were dissolved by adding 5% TCA. The samples (1 μl) were spotted on a silica gel plate (Silica gel 60F254, Merck) and developed with an acetate solution (ethyl acetate: acetic acid: water = 3: 3: 1). The plate was sprayed with aniline-diphenylamine-acetone-phosphoric acid solution (aniline: diphenylamine: acetone: phosphoric acid = 2 ml: 2 g: 100 ml: 15 ml) and heated to detect the spots.

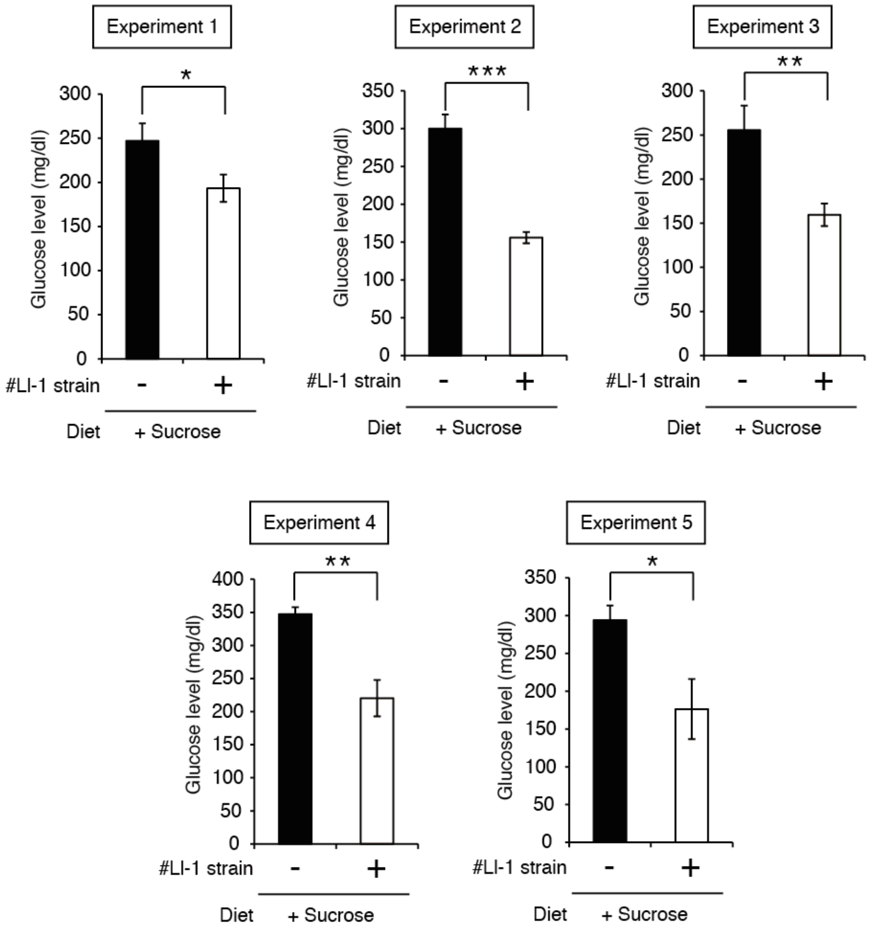

Supplementary figure 2 The inhibitory effects of *Lactococcus lactis* #Ll-1 strain against dietary sucrose-induced increases in silkworm hemolymph glucose levels. Silkworms were fed a diet containing 10% (w/w) sucrose or glucose with or without *Lactococcus lactis* #Ll-1 strain (25% [w/w] in diet) for 1 h. Glucose levels in the silkworm hemolymph were measured (n = 5/group). Data represent mean ± SEM. Significant differences between groups were evaluated using Student's *t*-test. * : $P < 0.05$, ** : $P < 0.01$. *** : $P < 0.001$.

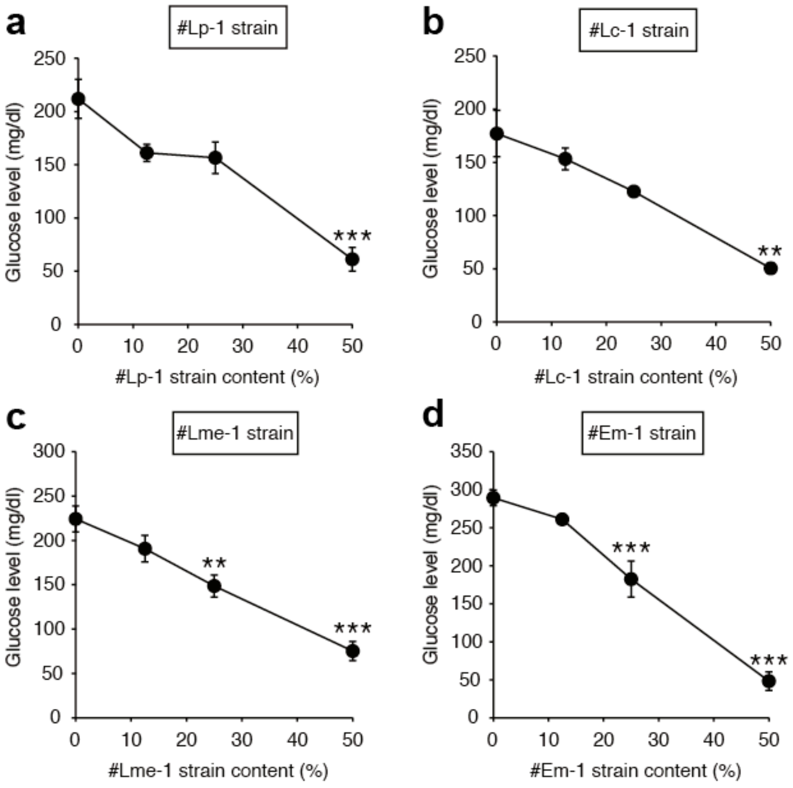

Supplementary figure 3 The inhibitory effects of the lactic acid bacteria strains against dietary sucrose-induced increases in silkworm hemolymph glucose levels. (**a**-**d**) Silkworms were fed a diet containing 10% (w/w) sucrose with or without the *Lactobacillus plantarum* #Lp-1 strain (**a**), the *Leuconostoc carnosum* #Lc-1 strain (**b**), the *Lactobacillus mesenteroides subsp. mesenteroides* #Lme-1 strain (**c**), and the *Enterococcus mundtii* #Em-1 strain (**d**) (0-50% [w/w] in diet) for 1 h. Glucose levels in the silkworm hemolymph were measured (n = 5/group). Data represent mean ± SEM. Significant differences between groups were evaluated using Student's *t*-test. ** : $P < 0.01$. *** : $P < 0.001$.

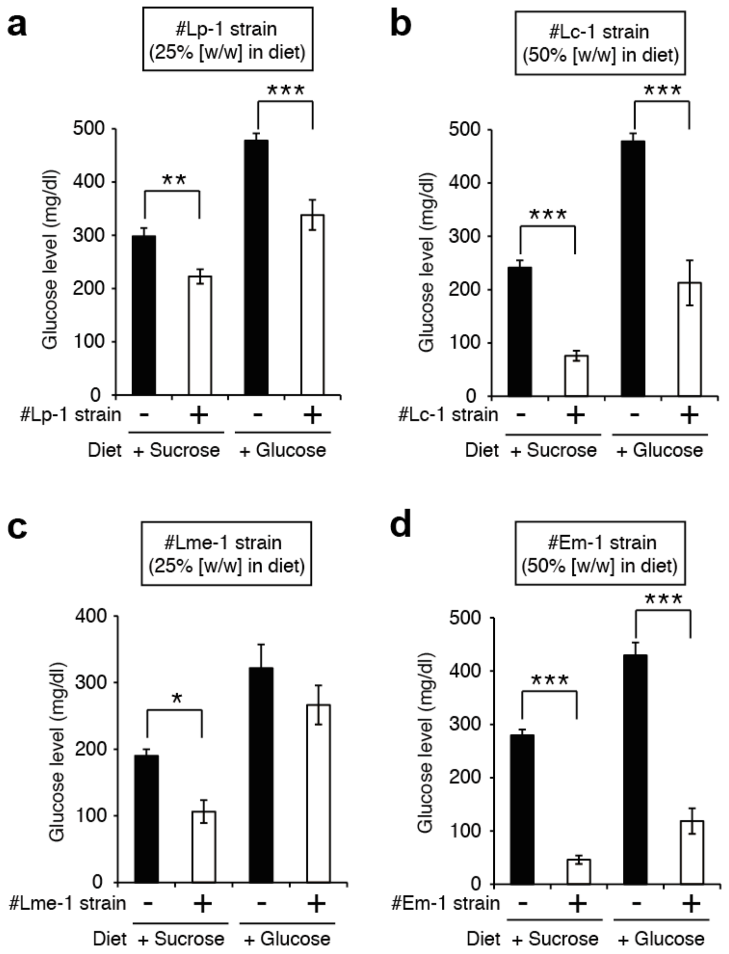

Supplementary figure 4 Effects of the lactic acid bacteria strains against dietary sucrose or glucose-induced increases in silkworm hemolymph glucose levels. (**a-d**) Silkworms were fed a diet containing 10% (w/w) sucrose or glucose with or without the *Lactobacillus plantarum* #Lp-1 strain (25% [w/w] in diet) (**a**), the *Leuconostoc carnosum* #Lc-1 strain (50% [w/w] in diet) (**b**), the *Lactobacillus mesenteroides subsp. mesenteroides* #Lme-1 strain (25% [w/w] in diet) (**c**), and the *Enterococcus mundtii* #Em-1 strain (50% [w/w] in diet) (**d**) for 1 h. Glucose levels in the silkworm hemolymph were measured (n = 5-7/group). Data represent mean ± SEM. Significant differences between groups were evaluated using Student's *t*-test. * : $P < 0.05$, ** : $P < 0.01$. *** : $P < 0.001$.

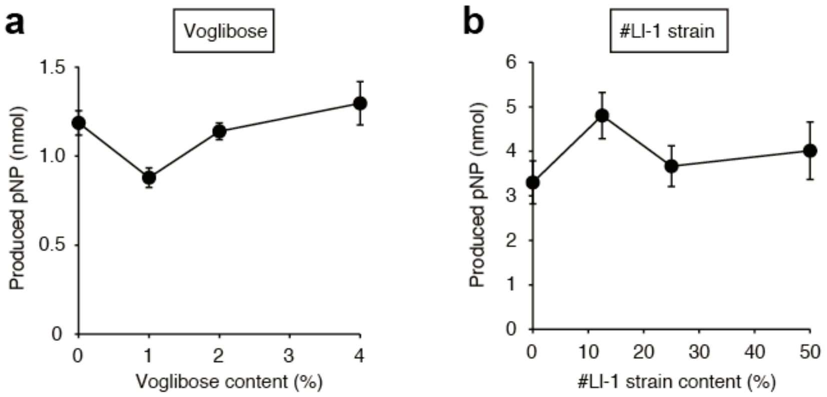

Supplementary figure 5 Effects of voglibose and #L1-1 strain against α-glycosidase activity in silkworm intestine. (**a**, **b**) Silkworms were fed a diet containing 10% (w/w) sucrose with or without voglibose or #L1-1 strain for 1 h. Silkworm intestines were collected and rinsed with PBS. The isolated intestine was disrupted by sonication, and a crude lysate of the intestine was used for analysis. The α-glycosidase activity per 1 μg of protein in the silkworm intestines was measured (n = 3-5/group). Data represent mean ± SEM.

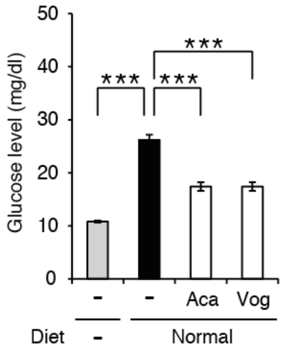

Supplementary figure 6 The inhibitory effects of acarbose and voglibose on dietary-induced increases in glucose levels in silkworm hemolymph. Silkworms were fed a normal diet (Normal diet) with or without acarbose (8% [w/w] in diet) or voglibose (4% [w/w] in diet) for 1 h. Glucose levels in the silkworm hemolymph were measured (n = 5/group). Aca: acarbose. Vog: voglibose. Data represent mean ± SEM. Statistically significant differences between groups were evaluated using Student's *t*-test. *** : $P < 0.001$.
